# Effect of crude degummed canola oil and *ad libitum* grazing on plasma metabolites of primiparous Holstein-Friesian cows in a pasture-based system

**DOI:** 10.1186/s12917-014-0224-5

**Published:** 2014-09-26

**Authors:** John R Otto, Bunmi S Malau-Aduli, Razaq O Balogun, Peter Nish, Aduli E O Malau-Aduli

**Affiliations:** Animal Science and Genetics, Tasmanian Institute of Agriculture, School of Land and Food, Faculty of Science, Engineering & Technology, University of Tasmania, Private Bag 54, Hobart, TAS 7001 Australia; College of Medicine and Dentistry, Division of Tropical Health and Medicine, James Cook University, Townsville, Queensland 4871 Australia; CopRice Feeds, PO Box 104, Cobden, Victoria 3266 Australia; TasHerd Pty Limited, P. O. Box 68, Hadspen, Tasmania 7290 Australia; Veterinary and Biomedical Sciences, College of Public Health, Medical and Veterinary Sciences, Division of Tropical Health and Medicine, James Cook University, Townsville, Queensland 4811 Australia

**Keywords:** Primiparous Holstein-Friesians, Crude degummed canola oil, Supplement, Plasma metabolites

## Abstract

**Background:**

The supplementation of fat to lactating dairy cows has long been used as a management tool to increase dietary energy density for improving cow production, reproduction and to alleviate negative energy balance. Attempts have been made to investigate the effect of canola meal on plasma metabolites in lactating cows, but the results have been diverse and inconsistent. To our current knowledge, there is a dearth of published information on the utilization of Crude Degummed Canola Oil (CDCO) in pasture-based dairy systems. Therefore, the objective of this study was to investigate the changes in plasma metabolite profiles of pasture-based, primiparous, Holstein-Friesian cows supplemented with varying dietary levels of CDCO for eight weeks. The study tested the hypothesis that *feeding grazing primiparous Holstein-Friesian cows for eight weeks with incremental levels of CDCO supplement will decrease plasma non-esterified fatty acids (NEFA) and β-hydroxybutyrate (BHBA), but increase plasma cholesterol and glucose metabolites.*

**Results:**

Twenty lactating primiparous Holstein-Friesian cows 40 days in milk were randomly allotted into four treatment groups that consisted of a wheat-based, pelleted basal diet with no supplemental CDCO (control), basal diet with CDCO added at 25 ml/kgDM (DM; dry matter) (low), 35 ml/kgDM (medium) and 50 ml/kgDM (high) in an eight-week feeding trial, after two weeks of adjustment. Treatment influenced BHBA but had no effect on plasma NEFA, cholesterol and glucose metabolite profiles (P > 0.05). However, week of supplementation had a significant effect (P < 0.05) on BHBA, NEFA and glucose concentrations.

**Conclusions:**

We concluded that with the exception of BHBA, CDCO at current levels of supplementation does not alter the plasma metabolite profiles of grazing primiparous cows. The lack of significant differences across treatments seems to indicate that higher levels of CDCO than the current levels used in this study, are probably needed. Furthermore, the duration of supplementation with CDCO had a greater impact on plasma metabolites than the levels of supplementation. Our findings also suggest that primiparous cows grazing high quality pastures during spring have sufficient energy intakes to prevent negative energy balance at 40 days in milk without the need for added fat supplements.

## Background

Primiparous Holstein-Friesian cows are the most energy-challenged animals on a typical pasture-based dairy farm, because in such a herd, they are at the bottom of the social hierarchy [1]. This is because primiparous cows are always the last to be milked and by implication, arrive last in the paddock, thus potentially reducing grazing time. This pattern, coupled with incidences of bullying and competition for grass in the paddock, contributes to low feed intake. Given that most first-time calvers are still heifers that are not fully grown at the time of calving (85 -90% of mature cow size), they have to regain post-partum weight loss (up to 100 kg of pre-calving weight) and also continue to grow and produce milk [1]. Therefore, primiparous cows tend to suffer more negative energy balance (NEBAL) than all the animals in the herd. With all these pressures and mating occurring fairly soon after calving, it is no wonder that they tend to have diminished milk production and reproduction performances.

**Figure 1:**
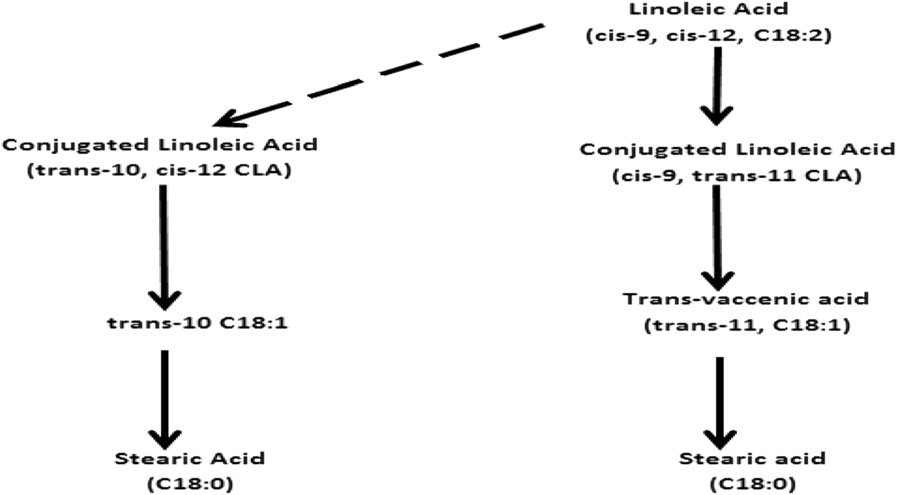
**Biohydrogenation of linoleic acid to stearic acid.**

Canola plant has been engineered to produce oil with greater concentrations of omega-9, omega-6 and adequate omega-3 fatty acids. Metabolism of canola oil in the rumen is facilitated by rumen microorganisms, particularly bacteria and protozoa. Bacterial lipase hydrolyses the triacylglycerols and phospholipids in the consumed dietary oil. Once the fatty acids are liberated from their ester linkages, the end products (glycerol and NEFA) are utilised in the biohydrogenation process. Biohydrogenation is an extensive microbial process that involves the addition of hydrogen molecules to unsaturated free fatty acids concentrated in the rumen. During biohydrogenation, unsaturated fatty acids (linoleic and alpha-linolenic acids) are extensively hydrogenated to form saturated fatty acids (stearic acid 18:0 and palmitic acid 16:0). The biohydrogenation of linoleic acid to stearic acid is demonstrated in Figure 1 [2].

Following biohydrogenation, the saturated and unsaturated fatty acids that escape this process are subsequently absorbed in the small intestine. As a result of rumen biohydrogenation, approximately 85% and 15% saturated and free fatty acids respectively, are transported into the small intestine and this process illustrates the efficiency of rumen microbes. Rumen biohydrogenation is the major factor affecting the delivery of fat in the small intestine and subsequent transportation in the blood of ruminants. Fat consumption by cows leads to the productions of volatile fatty acids (mainly acetate, butyrate and propionate). These volatile fatty acids are the precursors for the production of glucose, carbohydrates and fats.

The energy status of a cow is mostly reflected by its plasma non-esterified fatty acids (NEFA) and β-hydroxybutyrate (BHBA) profiles [3-6]. A reduction in the level of plasma glucose can also be used as an indicator of NEBAL in cows. Previous studies have suggested that NEFA concentration should be less than 0.2 mmol for normal cows [7]. However, values ranging from 0.5 to 0.7 mmol postpartum are indicative of NEBAL [8]. To prevent NEBAL in lactating cows, supplementation with limited amount of dietary fat in a pasture setting to boost postpartum energy intake has been of increasing research interest [9]. The effect of dietary fat supplements on plasma metabolites in dairy cows has been inconsistent and highly variable in the published literature. For instance, some studies found increased glucose, NEFA, BHBA and cholesterol [10-13], while others found no change [14-17] or had inconsistent results [18]. This suggests that further studies in different production systems are required to enable informed choices and tailored decisions when feeding lactating cows with specific dietary fat supplements, hence the justification for our study in a typical Australian pasture-based dairy production system.

The Australian dairy industry has increasing interest in crude degummed canola oil (CDCO) because of its ease of local availability and affordability. However, limited information currently exists in the published literature on the effect of CDCO on plasma metabolites. Therefore, this study intends to fill this knowledge gap by investigating the effect of dietary inclusion of CDCO at incremental levels for eight weeks on the plasma metabolite profiles of primiparous Holstein-Friesian cows in a pasture-based system. We hypothesized that *feeding grazing primiparous Holstein-Friesian cows for eight weeks with incremental levels of CDCO supplement will decrease plasma NEFA and BHBA, but increase cholesterol and glucose levels.*

## Results

### Effect of CDCO level and week of supplementation on plasma metabolites

Crude Degummed Canola Oil supplementation level had no significant differences on plasma NEFA compared to the control treatment. However, week of supplementation had a highly significant effect (P < 0.05) on plasma NEFA. Also, no significant interaction effect of treatment by week was detected on plasma NEFA (P > 0.05; Table 1).

**Table 1:** **Fixed and interaction effects (p-values) of CDCO treatment and week of supplementation on plasma metabolites**

**Effect**	**Plasma metabolites**			
	**BHBA**	**Cholesterol**	**Glucose**	**NEFA**
TRT	0.0241*	0.6681^NS^	0.4143^NS^	0.1314^NS^
Week	0.0001***	0.9415^NS^	0.0005**	0.0001***
TRT*Week	0.6489^NS^	0.9962^NS^	0.2613^NS^	0.8714^NS^

Level of significance: ^NS^ not significant (P > 0.05), *significant (P < 0.05), **highly significant (P < 0.01), ***very highly significant (P < 0.001); β-hydroxybutyrate (BHBA, mmol), non-esterified fatty acid (NEFA, mmol), Cholesterol (mmol), Glucose (mmol); Crude degummed canola oil (CDCO).

Plasma BHBA was significantly affected by both treatment (P < 0.0241) and week of supplementation (P < 0.05), although their interaction (treatment by week), was not significant (P > 0.05; Table 1). However, plasma BHBA concentration in cows receiving the high treatment was similar to that of cows in the control group (0.5 ± 0.0 vs 0.5 ± 0.0 mmol), but differed from those of cows receiving low (0.4 ± 0.0 mmol) and medium (0.4 ± 0.0 mmol) levels of CDCO (Table 2).

**Table 2:** **Least square means (LSM ± S.E) of plasma metabolites as influenced by CDCO treatment and week of supplementation**

**Effect**	**Plasma metabolites**			
	**BHBA**	**Cholesterol**	**Glucose**	**NEFA**
Control	0.5 ± 0.0^a^	5.8 ± 0.2	3.9 ± 0.1	0.2 ± 0.0
High	0.5 ± 0.0^a^	5.6 ± 0.3	3.9 ± 0.1	0.2 ± 0.0
Low	0.4 ± 0.0^b^	5.3 ± 0.3	3.7 ± 0.2	0.1 ± 0.0
Medium	0.4 ± 0.0^b^	5.5 ± 0.2	3.9 ± 0.1	0.2 ± 0.0
Week				
0	0.4 ± 0.0	5.5 ± 0.2	4.1 ± 0.1	0.2 ± 0.0
2	0.6 ± 0.0	5.6 ± 0.2	4.0 ± 0.1	0.1 ± 0.0
3	0.4 ± 0.0	5.6 ± 0.3	3.8 ± 0.1	0.2 ± 0.0
5	0.6 ± 0.0	5.5 ± 0.3	4.0 ± 0.1	0.1 ± 0.0
7	0.4 ± 0.0	5.3 ± 0.5	3.5 ± 0.2	0.2 ± 0.0
8	0.4 ± 0.0	5.7 ± 0.4	3.7 ± 0.1	0.1 ± 0.0

Column means within a variable bearing different superscripts significantly differ (P < 0.05); β-hydroxybutyrate (BHBA, mmol), non-esterified fatty acid (NEFA, mmol), Cholesterol (mmol), Glucose (mmol); Crude degummed canola oil (CDCO). Week 0, week before supplementation, week 2, second week of fat supplementation.

There were no significant (P > 0.05) differences in the mean plasma cholesterol and glucose concentrations of supplemented and unsupplemented cows. However, week of supplementation influenced glucose significantly (P < 0.05) as the level fell down in Week 2, but cholesterol was not affected (P > 0.05; Table 1).

### Correlations between traits

Table 3 shows that there were highly significant correlations (*P* < 0.001) between BHBA and NEFA (−0.32), cholesterol (0.24) and glucose (0.34). All other correlations were not significant (P > 0.05).

**Table 3:** **Pearson’s correlation coefficients between plasma metabolites**

**Traits**	**BHBA**	**Cholesterol**	**Glucose**	**NEFA**
BHB(mmol)		0.24*	0.34***	−0.32***
Cholesterol(mmol)	0.24*		0.25**	−0.02^NS^
Glucose(mmol)	0.34***	0.25**		0.03^NS^
NEFA(mmol)	−0.32***	−0.02^NS^	0.03^NS^	

Level of significance: ^NS^ not significant (P > 0.05), *significant (P < 0.05), **highly significant (P < 0.01), ***very highly significant (P < 0.001); β-hydroxybutyrate (BHBA), non-esterified fatty acid (NEFA).

### Weekly trends in plasma metabolites of supplemented and unsupplemented cows

**Figure 2:**
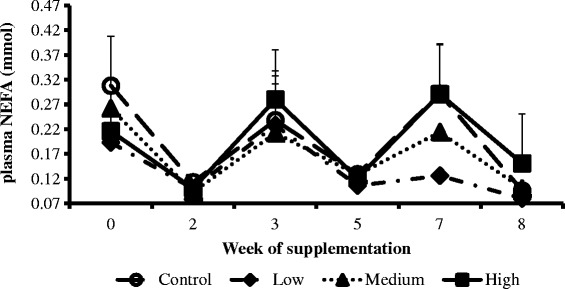
**Interaction between CDCO supplementation level and week of supplementation on plasma concentrations of non-esterified fatty acids.** Error bars (±SEM). Each treatment group had five cows. Week 0, week before fat supplementation, week 1, when fat supplementation commenced.

**Figure 3:**
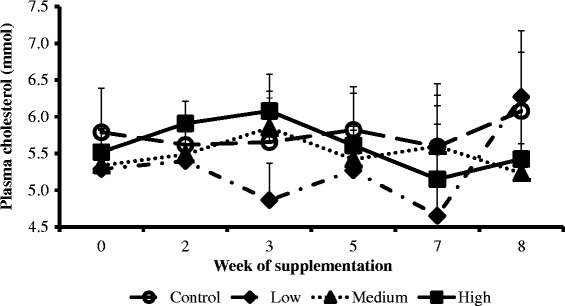
**Interaction between CDCO supplementation level and week of supplementation on plasma concentrations of cholesterol.** Error bars (±SEM). Week 0, week before fat supplementation, week 1, when fat supplementation commenced.

**Figure 4:**
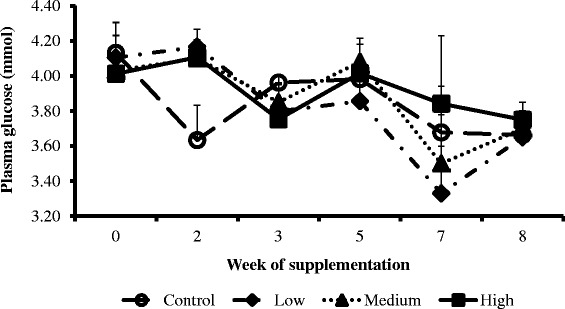
**Interaction between CDCO supplementation level and week of supplementation on plasma concentrations of glucose.** Error bars (±SEM). Week 0, week before fat supplementation, week 1, when fat supplementation commenced.

**Figure 5:**
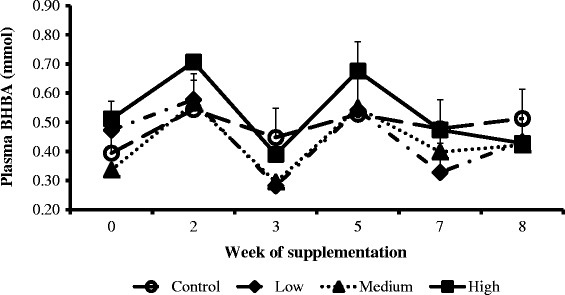
**Interaction between CDCO supplementation level and week of supplementation on plasma concentrations of β-hydroxybutyrate.** Error bars (±SEM). Week 0, week before fat supplementation, week 1, when fat supplementation commenced.

The weekly concentration trends of NEFA (Figure 2), cholesterol (Figure 3) and glucose (Figure 4) were similar across treatments and the control groups. However, the weekly BHBA trends for cows in the high group were higher compared to the medium, low and control groups (Figure 5).

## Discussion

Enormous remobilization of NEFA from adipose tissues postpartum occurs in high merit cows as a sign of transition from gestation to milk production [8]. However, high concentrations of NEFA in the blood can be toxic and compromise the production and reproduction performances of cows [19-22]. Plasma NEFA can provide the body tissues with energy when not in excessive quantities. However, when the animal is suffering from negative energy balance, excessive amounts of NEFA in the blood can be toxic to the animal. Most of the NEFA in the liver can be metabolised into triacylglycerols. Accumulated NEFA leads to excess triacylglycerols accumulating in the liver and the unutilised residual acetyl-CoA from the oxidation of fatty acids in the tricarboxylic acid cycle is converted into ketone bodies, such as acetone, acetoacetate and BHBA. The results of our current study found no significant effect of feeding CDCO on NEFA concentration in contrast to Andersen et al. [23] and Douglas et al. [24] who reported increases in NEFA. These previous studies utilized different forms and higher dosages of dietary fat sources which could explain the observed differences. The CDCO levels utilised in our study were tailored to avoid feeding greater than the 7% total fat allowed in the diets of grazing animals [9] because it can affect dry matter intake and ruminal fibre digestion [9]. Another explanation for the lack of observable differences between the supplemented and unsupplemented groups of animals in our study could be related to the metabolisable energy component of the CDCO which was very similar for both the control and basal diet (isocaloric). Secondly, the result also suggests that the ryegrass pastures that were grazed by the cows were of high quality and provided adequate energy to the postpartum cows to prevent massive adipose tissue remobilisation. The similarity across weeks and between treatment groups (average 0.2 mmol) in plasma NEFA suggests similar energy intake and utilisation. It has been suggested that three weeks pre-partum to three weeks post-partum is the most energy deficit period for high merit cows [25], but the cows in our study were already 40 DIM (days in milk) and might have well passed the critical energy deficit periods, hence the lack of observable treatment differences. It has also been reported that the level of plasma NEFA was greater in primiparous cows soon after calving [26], implying that fat supplementation studies on NEFA should be carried out during the early calving period [27].

Plasma BHBA is a product of NEFA that is converted into triacylglycerols in the liver [28,29]. In the present study, feeding CDCO significantly influenced plasma BHBA concentration where a slight, but significant increase in BHBA was observed as the level of CDCO increased from low to high (Table 2). Our results are in contrast with those of [30] who found that when rumen inert fat was fed to dairy cows, plasma BHBA decreased prepartum. This disparity could be linked to the differences in the physiological states of the experimental cows. In our study, the cows were only 40 days in milk coinciding with the early lactation phase, while in their study, the cows were 300 days in milk almost at the end of their lactation. Another major area of difference between the two studies lies in the type of fat fed. While we supplemented the cows with crude degummed canola, they fed an inert fat that is rumen-protected. The effect of fat supplementation on plasma BHBA has been associated with the availability of carbohydrates [31] and the impact of long chain fatty acids (particularly Docosahexaenoic acid, C22:6) on hepatic gluconeogenesis [32]. It would thus appear that in our study, as the level of CDCO supplementation increased, hepatic gluconeogenesis also increased due to a likely influx of available dietary carbohydrates and docosahexaenoic acid.

Cholesterol contains lipoproteins and the high and low density lipoproteins [33-35], which are all precursors for progesterone metabolism [35]. Progesterone is one of the hormones essential for fertility in cows. Fat supplements have been used efficiently to alter the plasma cholesterol concentration of dairy cows [36,37]. However, in the current study, dietary fat did not influence plasma cholesterol concentration. This can partly be explained by the fact that canola is known to contain mainly phytosterol [38,39]. Phytosterol contains low levels of cholesterol [38]. Previous studies have also reported that phytosterol can significantly reduce cholesterol in humans with hypercholesterolemic condition [40]. Therefore, the lack of significant effect observed in the present study could be explained by the hypocholesterolemic effect of CDCO.

The demand for glucose rises sharply post-partum due to increased energy requirements for lactation [7]. However, due to low dry matter intake after parturition, the amount of glucose produced is not enough to support the cow’s lactation requirements. Ruminal propionate produced during ruminal fermentation is used as a substrate in the gluconeogenesis pathway to produce glucose [41-43]. The effect of dietary fat supplements on plasma glucose has been associated with their ability to provide adequate rumen propionate [42,43]. In the current study, there were no significant differences between CDCO-supplemented and unsupplemented cows. The lack of significance in the present result indicates that the dietary fat treatment supplied adequate propionate to the rumen to affect plasma glucose concentration. Additionally, the effect of biohydrogenation of unprotected supplementary fat in the rumen was not any different between supplemented and control group of cows [44,45], hence the availability and levels of substrates necessary for glucose metabolism were similar in all cows.

Week of supplementation had a significant influence on NEFA, BHBA and glucose. This seems to suggest that the longer cows are supplemented with CDCO, the greater the impact on plasma metabolite profiles. The negative correlation between NEFA and cholesterol, and the positive correlation between cholesterol and glucose in the present study corroborate the theory that negative energy balance can impact negatively on production parameters in a pasture based setting.

When plasma glucose levels decrease, body fat remobilization is instigated from nutrient accrual to provide sufficient energy that can maintain continuous milk production until the animal returns to a positive energy balance. It has been found that cows suffering from negative energy balance have increased concentrations of serum glucagon and growth hormones, whereas the concentrations of insulin and insulin growth factor-I are decreased. Some proposed theories postulate that dietary fat supplement favours lower blood NEFA concentration by providing extra energy postpartum. Other research findings indicate that feeding dairy cows with fat supplements could promote increased insulin production because of the amount of increased energy provided through the production of acetate, propionate and butyrate (precursors for glucose, fat and carbohydrate production). However, studies investigating the response of plasma insulin to fat supplementation are inconsistent. For instance, some studies reported decreased plasma concentrations of insulin, while others reported steady insulin increases postpartum in cows fed six different diets containing fats. Therefore the mechanism of fat supplementation and relationship with insulin production is still poorly understood and warrants further elucidation.

## Conclusions

Canola supplements are effective dietary energy sources because they are involved in the production of volatile fatty acids which serve as lipid metabolic substrates for the synthesis of glucose, fats and carbohydrates for lactation. The concentrations of plasma NEFA, BHBA and glucose are indicators for gauging the energy balance status of a lactating cow. Week of supplementation was a more significant factor than level of CDCO supplementation in influencing plasma metabolite profiles, thus suggesting that the duration of supplementation with CDCO has a greater impact on all the plasma metabolites investigated in this study. It was also apparent from this study that primiparous cows grazing high quality pasture at about 40 days in milk (DIM), had adequate energy intake to overcome any extreme negative energy balance scenario at this stage of lactation. It also implies that fat supplementation may not be necessary in spring when there is abundant and lush pasture, but may be needed during winter or summer when pasture is scanty to boost the energy intake of cows. The hypothesis that *feeding grazing primiparous Holstein-Friesian cows for eight weeks with incremental levels of CDCO supplement will decrease plasma non-esterified fatty acid (NEFA) and β-hydroxybutyrate (BHBA), but increase plasma cholesterol and glucose metabolites* does not hold true and should be rejected. Therefore, it is concluded that primiparous Holstein-Friesian dairy cows in a pasture-based setting have enough energy intakes from grass in spring to maintain adequate production and reproduction performances. However, there is the need for further investigation into the interaction between circulating plasma hormones and gene expression profiles of supplemented cows to provide a better understanding of CDCO’s role in future applications as a dietary fat supplement for lactating cows.

## Methods

### Site and climatic conditions

All experimental procedures were in accordance with the University of Tasmania Animal Ethics Committee guidelines (Animal Ethics Permit Number A0012583), the 1993 Tasmania Animal Welfare Act and the 2004 Australian Code of Practice for the Care and Use of Animals for Scientific Purposes. The experiment was carried out at the University of Tasmania’s Dairy Research Centre, Tasmanian Institute of Agriculture (TIA) Elliot Dairy Research Farm in Somerset, North-Western Tasmania, Australia, from September to November 2012. Tasmania is Australia’s smallest state with a land size of 68,000 square kilometers and located within the cool, temperate, climatic zone at latitude 42° South and longitude 147° East. It is characterized by four distinct seasons - winter, autumn, spring and summer. The experiment was carried out in spring when the annual rainfall was 2500 mm and humidity was approximately 60%.

### Animals and treatments

The condition and energy status of the experimental cows was visually assessed based on body condition score (BCS) on a scale of 1–5 [46,47], the cows were in negative energy balance. Twenty primiparous, spring-calving, purebred, Holstein-Friesian cows (average liveweight of 400 ± 40 Kg, BCS 4 ± 1, 40 ± 8 days in milk (DIM) early lactation phase; and daily milk yield of 20.7 litres), were randomly allocated into 1 of 4 treatments of CDCO (25 ml/KgDM, 35 ml/KgDM and 50 ml/KgDM) and the control (no CDCO- 0 ml/KgDM). This replicated herd of cows (n = 5 per treatment group) receiving CDCO supplements was placed under the same management and rotated in electric fenced paddocks with the Control cows offered wheat-based pellets without CDCO. Together, the animals had access to 3000 kg DM of forages, a mixture of ryegrass (*Lolium perenne*), cocksfoot (*Dactylis glomerata*), and white clover (*Trifolium repens)* pasture grazed at the two-leaf stage. Water was offered at *ad libitum.* The treated cows grazed the same pasture allotment as the Control cows but were offered CDCO plus wheat-based pellet at the rate of 50 ml/KgDM (for the high level of supplementation group), 35 ml/KgDM (medium level of supplementation group) and 25 ml/KgDM (low level of supplementation group). The current level of CDCO was calculated based on 7% total fat allowed in the diet of grazing cows [9] and the physiological status of being in the early lactation phase. Each cow received 6 kg of the pelleted supplements daily for eight weeks, after two weeks of adjustment. Supplements were offered to cows in two splits; morning (3 kg) and evening (3 kg) milking sessions at 0500 h and 1500 h. There was no feed residual left over from any of the groups. The exact pasture intake was difficult to estimate as the case is under grazing conditions. The chemical compositions of the treatment, control and basal feeds are presented in Table 4.

**Table 4:** **Chemical composition of feed components**

**Component (%DM)**	**Feed components**		
	**Treatment feed (canola oil)**	**Control feed (No canola oil)**	**Basal diet (Pasture)**
MC	8.2	9.1	55.0
DM	91.8	90.9	94.5
ADF	8.0	9.0	27.7
NDF	20.0	21.1	45.9
EE	6.2	2.1	3.0
Ash	9.7	8.9	9.3
NFC	52.8	59.0	23.9
CP	12.7	10.4	21.0
ME (kJ/100gDM)	4083.3	4065.7	3999.2

All feeds were analyzed on a dry weight basis; Moisture content (MC), Dry matter (DM), organic matter (OM), neutral detergent fiber (NDF), acid detergent fiber (ADF), non-fibrous carbohydrate (NFC), ether extract (EE), crude protein (CP) and metabolisable energy (ME).

### Feed chemical composition and analysis

Dry matter (DM) content of the basal and experimental diets was determined by drying samples to a constant temperature at 65°C in a fan forced oven, finely ground to pass through a 2 mm sieve using Laboratory Mill (^a^Thomas Model 4 Wiley® Mill; Thomas Scientific), and further drying at 105°C for 24 h. The DM was computed as the difference between the initial and final weights of samples expressed as a percentage. Moisture content was determined by subtracting the% DM from 100%. Ash content was determined by combusting samples in a furnace at 600°C for 8 hours. Neutral detergent (NDF) and acid detergent fiber (ADF) contents were measured using an Ankom fiber analyzer, ^b^ANKOM^220^; ANKOM Technology, USA [48]. The analysis for total nitrogen was determined using a ^c^Thermo Finnigan EA 1112 Series Flash Elemental Analyzer [49] and the values multiplied by 6.25 to give the crude protein (CP) percentage. Ether extract (EE) was determined using an Ankom fat/oil extractor (^d^ANKOM^XT15^; ANKOM Technology, USA) based on hexane-petroleum ether solvent extraction. Metabolisable energy (ME) was calculated as per Weiss [50].

### Blood sample collection and plasma metabolite analysis

Blood samples (10 ml) were collected from each experimental cow after the morning milking (0500 h) on week zero and fortnightly thereafter, until the end of the experiment. All samples were collected by coccygeal venipuncture into heparin vacutainers. All collected blood samples were centrifuged at 1,125 X g for 10 minutes at 4°C to facilitate distinct separation between the plasma and serum. The plasma fractions were decanted into 2 ml vials, sealed with an airtight cap and stored at −20°C until further laboratory analyses. Plasma NEFA, BHBA, cholesterol and glucose samples were commercially analysed at the Animal Health Laboratories, Department of Agriculture and Food (South Perth, Australia) using appropriate kits (ACS-ACOD Method) from Wako Pure Chemical Industries Ltd (Code No. 279–75401) on ^e^Beckman Coulter (AU 400) analyzer.

### Statistical analysis

Initially, summary statistics by level and week of CDCO supplementation were computed to give means, standard deviations standard error, variance, minimum and maximum values that were scrutinised for any data entry errors. Testing for linear, quadratic and cubic orthogonal contrasts by regressing the dependent on explanatory variables was carried out using PROC REG (SAS 2009). Subsequently, NEFA, BHBA, glucose and cholesterol were analysed by repeated measures analysis of variance using PROC MIXED (SAS , 2009) utilising compound symmetry covariance structure and week of supplementation as the repeated effect. The model included treatment, week of lactation and interaction between treatment and week of lactation as fixed effects, while base line milk values and cows were fitted as random effects and the degrees of freedom were estimated by the Satterthwaite method (SAS, 2009). Variables of interest having significant treatment and or week of lactation effects are presented in Tables and Figures as pooled Least Squares Means and Standard Error of Means (LSM ± SEM) and differences between means were considered significant at the *P* < 0.05 threshold unless otherwise stated. Significant differences and mean separations were carried out using Tukey’s probability pairwise comparison tests (SAS, 2009). Pearson correlation coefficients between dependent variables were estimated using PROC CORR (SAS, 2009) with significance determined using Bonferroni’s probability pairwise comparison test (SAS, 2009). Correlation analyses were initially carried out on the whole data set and also by week of supplementation, but the weekly correlation values were dropped and those from the whole data retained because there were no significant differences between the two sets of values.

## Endnotes

^a^Thomas Model 4 Wiley® Mill; Thomas Scientific.

^b^ANKOM^220^; ANKOM Technology, USA.

^c^Thermo Finnigan EA 1112 Series Flash Elemental Analyzer.

^d^ANKOM^XT15^; ANKOM Technology, USA.

^e^Beckman Coulter (AU 400) analyzer.

## Abbreviations

ADF: Acid detergent fiber
BHBA: β-Hydroxybutyrate
CDCO: Crude degummed canola oil
CP: Crude protein
DM: Dry matter
EE: Ether extract
ME: Metabolisable energy
MC: Moisture content
NEBAL: Negative energy balance
NDF: Neutral detergent fiber
NEFA: Non-esterified fatty acids
NFC: Non-fibrous carbohydrate
OM: Organic matter
SAS: Statistical analysis software
